# Electroosmotic Flow of Non-Newtonian Fluid in Porous Polymer Membrane at High Zeta Potentials

**DOI:** 10.3390/mi11121046

**Published:** 2020-11-27

**Authors:** Shuyan Deng, Yukun Zeng, Mingying Li, Cuixiang Liang

**Affiliations:** 1 Institute of Architecture and Civil Engineering, Guangdong University of Petrochemical Technology, Maoming 525000, Guangdong, China; dshy530@sina.cn (M.L.); liang_cx@126.com (C.L.); 2 Institute of Mechanical and Electrical Engineering, Guangdong University of Petrochemical Technology, Maoming 525000, Guangdong, China; kun2000221@163.com

**Keywords:** electroosmotic flow, power-law fluid, porous polymer membrane

## Abstract

To help in the efficient design of fluid flow in electroosmotic pumps, electroosmotic flow of non-Newtonian fluid through porous polymer membrane at high zeta potentials is studied by mainly evaluating the total flow rate at different physical parameters. Non-Newtonian fluid is represented by the power-law model and the porous polymer membrane is considered as arrays of straight cylindrical pores. The electroosmotic flow of non-Newtonian fluid through a single pore is studied by solving the complete Poisson–Boltzmann equation and the modified Cauchy momentum equation. Then assuming the pore size distribution on porous polymer membrane obeys Gaussian distribution, the performance of electroosmotic pump operating non-Newtonian fluid is evaluated by computing the total flow rate of electroosmotic flow through porous polymer membrane as a function of flow behavior index, geometric parameters of porous membrane, electrolyte concentration, applied voltage, and zeta potential. It is found that enhancing zeta potential and bulk concentration rather than the applied voltage can also significantly improve the total flow rate in porous polymer membrane, especially in the case of shear thinning fluid.

## 1. Introduction

Microfluidic technologies have attracted increasing attention recently due to the broad applications in numerous fields such as microelectric mechanic systems, bio-sensor systems, and on-chip experimental systems [1,2,3]. The fluid flow encountered in micropumps and micromixers is essentially different from the flow in macro-scale. To optimize micromachines, the flow behavior in a microchannel has to be treated carefully. The contact between electrolyte solution and the wall surface of microchannel triggers exchange of charges, resulting in formation of electric double layer (EDL) [4,5]. When an electric field is applied along the central line of microchannel, the migration of free ions in EDL caused by the Coulombic force and the fluid viscosity generates the so-called electroosmotic flow (EOF). EOF has been the main flow form in the above applications because of the operational advantages over conventional pressure-driven flow such as high flow rate and smooth pulse-free flow with plug-like pattern [6].

Electroosmotic pump (EO pump) is the key actuator system in micromechanical devices such as drug delivery system, microelectronics equipment cooling, and biological analysis [7]. EO pump is able to control small amount of fluid precisely, also has no moving part and can be integrated on microchips [8,9,10]. Porous-media-based EO pumps have been greatly investigated because the flows in porous media can be treated as that through a large number of straight microchannels in parallel and hence can provide good pumping performance. Furthermore, pumping material plays an important role on the performance of EO pumps. For instance, the bead packing EO pump suffers from drawbacks, such as its tedious fabrication procedure and difficulty of anchoring beads at specific locations. Yao et al. designed and fabricated a porous glass structure EO pump in [11], the performance of which was predicted in [12] by solving the flow rate as a function of geometric parameters of membrane and chemical properties of bulk. Kim et al. investigated the performance of porous glass structure EO pump operating liquids with various ion density [13]. From the literature on the EO pump above, it is found that to pursue high flow rate and pressure capacity, increasing applied electric voltage is the most achievable method. However, high driving voltage can lead to Joule heating in electrolyte solution and in further lower efficiency of EO pump. Therefore, low-voltage (1–10 V) EO pumps were proposed in [14,15,16]. Among them, the thin membrane was actively used so that the length of microchannel namely the distance between the two driving electrodes is reduced and thus a relatively low electric voltage can also generate a high electric field. On the other hand, to improve the performance of EO pumps, polymer materials are increasingly attracting attention because of the low cost, high biocompatibility, and flexibility. Kwon et al. evaluated the performance of porous polymer membrane structure EO pump in the absence of back pressure and the comparison study between the performance of porous polymer membrane structure EO pump and porous glass structure EO pump shows that the porous track-etch polymer material excels over the porous glass largely due to the thickness and flexibility [17]. Compared to the porous materials used in low-voltage EO pump, the porous polymer membrane can bond with polymer material and facilitate a miniaturization procedure.

In EO pump applications, the working liquids have been represented as Newtonian model. However, it is necessary to incorporate the nonlinear constitutive relation of the non-Newtonian fluid because non-Newtonian fluids, such as biological samples or polymer solution with long-chain molecules, have been actively manipulated. Das and Chakrabotry [18,19] investigated the steady EOF of the inelastic power-law fluid. It turns out that the power-law constitutive relation models fluids in engineering applications very well. Thus, efforts have been put on electrokinetic flows of power-law fluids in varieties of microgeometries [20,21,22]. The EOF in a rectangular microchannel [23], in a cylindrical microcapillary [24], and the electroviscous effects of periodical pressure-driven flow [25] of power-law fluid in a rectangular microchannel have been studied in my previous works. In addition, most works associated with estimation of EO pumps assume the absolute value of zeta potential equal to 0.025 V or lower to simplify the model. According to measurements [12,17,26,27], the zeta potential in porous polymer membrane structure EO pumps [12,17] and in other practical applications [26,27] possibly can go beyond 0.025 V and even arrive at 0.2 V in which case Debye–Hückel approximation is unsuitable. By means of the approximation method proposed by Philip and Wooding [28], Levine et al. [29] studied the electrokinetic flow at high zeta potential at first. Kang et al. [30] focused on the dynamic aspect of EOF without using the Debye–Hückel approximation. It is found that the increase in zeta potential exerts significant influence on the flow rate of EOF. Therefore, in studies of EO pumps, a new model which incorporates those practical issues in EO pump is highly desired for precisely predicting performance of EO pump.

To the author’s knowledge, few theoretical works have been reported concerning the theoretical evaluation of EO pumps operating non-Newtonian fluid. The novelty of this paper is to study EOF of power-law fluids through porous polymer membrane at high zeta potentials in the context of EO pump applications. The complete Poisson–Boltzmann (P-B) equation and the modified Cauchy momentum equation for power-law fluid flow are solved to characterize the EOF of power-law fluids in a single pore of porous polymer membrane. Then with the assumption that the porous membrane has a Gaussian distribution of parallel cylindrical pores (such as the porous glass [11,12,14], porous polymer membrane [15,16,17]), the total flow rate through porous track-etch polymer membrane is computed as a function of flow behavior index, zeta potential, electric voltage, and concentration of solution. The prediction for the flow rate of EO pump shows qualitative agreement with the existing measured flow rate in [17]. It means the mathematical model and numerical method applied are valid. Correspondingly, the theoretical study carried out in this paper can predict performance of EO pump operating non-Newtonian fluid. Section 2 presents the mathematical formulation of EOF of non-Newtonian fluid through porous polymer membrane and the semi-analytical solution to the governing equations. In Section 3, the theoretical results including the electroosmotic transport characteristics in arbitrary single pore and the flow rate through porous polymer membrane at different physical parameters are presented. Section 4 draws a conclusion and provides guidance for evaluating EO pump performance. For EOF through polymer membrane, it is found that enhancing zeta potential and bulk concentration rather than the applied voltage can also significantly improve the total flow rate in porous polymer membrane, especially in the case of shear thinning fluid.

## 2. Problem Formulation

EOF of power-law fluid in porous media is described in terms of the electroosmotic transport characteristics in a single pore and the total flow rate in porous media. As shown in Figure 1 in [17], the porous polymer track-etch membrane with given surface area *A*_t_ is considered here. From the surface view in Figure 1 from [17], the arbitrary pore with radius *R* can be assumed as a straight cylindrical microcapillary and when an external electric field is applied along the cross section of porous membrane, the power-law fluid in porous membrane of EO pumps forms EOF and generates flow rate.

### 2.1. Mathematical Model for Electroosmotic Flow (EOF) of Power-Law Fluid in a Single Pore

EOF of power-law fluid through a single pore of porous polymer membrane is modelled at first. The arbitrary single pore is treated as a straight cylindrical microcapillary and sketched in.

The assumptions used to establish the mathematical model are presented as follows:

(1) According to microfluidic transport of liquid in microfluidic devices, the pore radius is much less than its length [1] and hence the flow velocity is assumed to be only along *z*-axis;
(2) Each single pore is filled by ideal symmetric electrolyte solution with constant permittivity and no interaction between ions occurs [26];
(3) The thickness of EDL is much less than the pore radius and EDLs will not overlap [26];
(4) The pore surface is charged with constant zeta potential [2,17];
(5) Gravitational force is negligible [2], no external pressure gradient is imposed and an electric field is applied over the cross-section of microchannel;
(6) An incompressible, laminar, and fully developed flow is assumed.

According to the electrostatic theory and Assumptions (1)–(4), P-B equation for the electric potential *ψ*(*r*) across a single pore is obtained

(1) $$\frac{1}{r}\frac{d}{dr}\left(r\frac{d\psi }{dr}\right)=\frac{2en_{\infty }}{\varepsilon_{r}\varepsilon_{0}}\sinh\left(\frac{e\psi }{k_{b}T}\right)$$

in which *ε_r_* is the relative permittivity, *ε*_0_ is the permittivity in vacuum, *n_∞_* is the ionic number concentration at the neutral state where *ψ*(*r*) = 0, *e* is the elementary charge, *k_b_* is Boltzmann constant and *T* is the absolute temperature [30].

The corresponding boundary conditions are

(2) $$r=0:\frac{d\psi }{dr}=0,r=R:\psi =\zeta$$

where *ζ* is the zeta potential. The ions are attached to surface of channel wall due to the absorption effect from the surface and thus immobile, which becomes inner layer of EDL with thickness of one diameter of one hydrated ion. The mobile cations and anions exist outside the inner layer is also known as diffuse layer or outer layer of EDL. An electric potential exists at the shear plane between inner layer and outer layer, which is called zeta potential.

**Figure 1:**
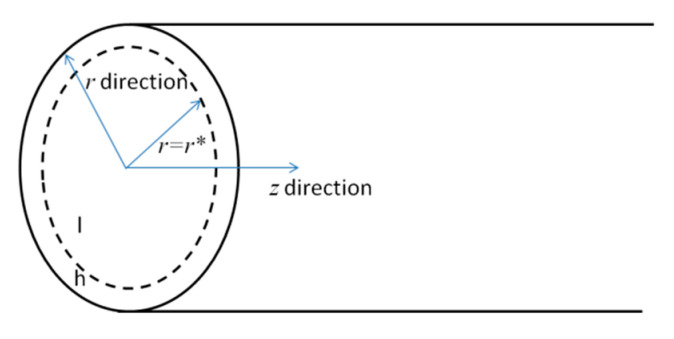
Coordinate system used for modelling EOF in each single pore of porous polymer membrane.

To circumvent the nonlinearity of Equation (1), an approximate solution to Equations (1) and (2) is proposed in [28] where the solution domain is comprised of low potential regime *l* and high potential regime *h* (see Figure 1) and the corresponding solutions for electric potentials are

(3) $$\psi_{l}\left(r\right)=\frac{k_{b}TI_{0}\left(r\right)}{eI_{0}\left(r^{*}\right)},0\leq r\leq r^{*}$$

(4) $$\psi_{h}\left(r\right)=\frac{k_{b}T}{e}\ln\left\{\frac{-C}{{\left(r\right)}^{2}\cos^{2}\left[B+\frac{1}{2}\sqrt{-C}\ln\left(\frac{r}{R}\right)\right]}\right\},r^{*}\leq r\leq R$$

where *ψ_l_*(r^*^) = *ψ_h_*(r^*^), *B* = cos^−1^[−*C*/(exp(*eζ*/*k_b_T*)(*R*)^2^)]^1/2^ and the integration constant *C* = [2 + r^*^I_1_(r^*^)/*I*_0_(r^*^)]^2^ − *exp*(1)·(r^*^)^2^ with *i*th-order modified Bessel function *I_i_*(*r*). The specific procedure can be found in [28].

The general form of motion equation for EOF is

(5) $$\rho_{m}\left(\frac{\partial \vec{V}}{\partial t}+\vec{V}\cdot \nabla \vec{V}\right)=-\nabla \vec{p}+\nabla \cdot \vec{\tau }+\vec{E}\rho_{e}$$

where *ρ_m_* represents the mass density, $\vec{V}$ represents EOF velocity, *t* denotes the time variable, *p* denotes the pressure and *E* denotes the electric velocity strength. For power-law fluids, the shear stress distribution is a non-linear function of velocity gradient. Namely, $\vec{\tau }=2m{\dot{\gamma }}^{n-1}\vec{e}$ is the shear stress distribution defined with $\dot{\gamma }={\left(2e_{kl}e_{kl}\right)}^{1/2}$ being the second invariant of the strain rate tensor, *e_ij_* = 1/2(∂*u_i_*/∂*x_j_* + ∂*u_j_*/∂*x_i_*), *n* denotes the flow behavior index and *m* refers to the flow consistency index with dimension [Nm^−2^s*^n^*]. Using Assumptions (1), (5) and (6), for a single direction EOF in each single pore of porous polymer membrane, the velocity is reduced to the component along the axial direction *u*(*r*) and thus the corresponding constitutive equation of power-law fluid can be written as *τ* = *m*(−*du/dr*)*^n^*^−1^·(*du*/*dr*). For the power-law model, the change in flow behavior index *n* is responsible for the change in viscosity and shear stress. *n* < 1, *n* = 1, and *n* > 1 correspond to shear thinning (pseudoplastic) fluid, Newtonian fluid, and shear thickening (dilatant) fluid, respectively. With the combination of Assumptions (5) and (6), substituting Equation (7) into Equation (6), the modified momentum equation can be obtained

(6) $$\frac{1}{r}\frac{d}{dr}\left[rm{\left(-\frac{du}{dr}\right)}^{n}\right]=\rho_{e}E$$

The boundary conditions that Equation (6) is subject to can be expressed as

(7) $$r=0:\frac{du}{dr}=0,r=R:u=0$$

In the case of Newtonian fluid (*n* = 1) and low zeta potential (|*ζ*| ≤ 0.025 V) where Debye–Hückel approximation can be applied [30], the analytical solution to Equations (6) and (7) is

(8) $$u\left(r\right)=\frac{\varepsilon_{r}\varepsilon_{0}\zeta E}{\mu_{0}}\left[\frac{I_{0}\left(kr\right)}{I_{0}\left(kR\right)}-1\right]$$

where *k* = (2 *e*^2^*n*_∞_/*ε*_r_*ε*_0_*k_b_T*)^1/2^ and 1/*k* denotes the thickness of EDL. Accordingly, the flow rate and average velocity of Newtonian fluid can be solved

(9) $$Q_{N}=\frac{2\pi R^{2}\varepsilon_{r}\varepsilon_{0}\zeta E}{\mu_{0}}\left[\frac{2I_{1}\left(kR\right)}{kRI_{0}\left(kR\right)}-1\right]$$

(10) $$U_{N}=\frac{2\varepsilon_{r}\varepsilon_{0}\zeta E}{\mu_{0}}\left[\frac{2I_{1}\left(kR\right)}{kRI_{0}\left(kR\right)}-1\right]$$

While in the case of non-Newtonian fluid (*n* ≠ 1) and high zeta potential (|*ζ*| > 0.025 V), the semi-analytical solution to Equations (6) and (7) can be solved

(11) $$u\left(r\right)={\left[{\left(-1\right)}^{n-1}\frac{2n_{\infty }eE}{\mu_{0}}\right]}^{1/n}{\int_{R}^{r}\left[\frac{1}{r_{2}}\int_{0}^{r_{2}}r_{1}\sinh\frac{e\psi \left(r_{1}\right)}{k_{b}T}dr_{1}\right]}^{1/n}dr_{2}$$

where *r*_1_ and *r*_2_ are temporary space variables. Equation (11) is solved using the numerical integral method, where the method of variable step discretization is applied along *r* and then each step is computed by Gauss numerical integration. After computing velocity profile *u*(*r*), the flow rate and average velocity for EOF of power-law fluids in a single pore is solved from the following expressions

(12) $$Q_{s}=2\pi \int_{0}^{R}r\cdot u\left(r\right)dr$$

(13) $$u_{s}=\frac{2}{R^{2}}\int_{0}^{R}r\cdot u\left(r\right)dr$$

### 2.2. Mathematical Model for EOF of Power-Law Fluid through Porous Polymer Membrane

The prediction of total flow rate through membrane in EO pump is an important step in EO pump operation and optimization. The applied electric voltage, zeta potential, geometric properties of porous polymer membrane, and ion density of electrolyte solution are adjustable parameters as estimating EO pumps, the effects of which are targeted in this study. A mathematical model for EOF of power-law fluid in porous materials in EO pumps such as hydra gel (porous polymer membrane: porous polycarbonate track-etch membranes) [17] is presented by extending the model of EOF in a single pore. The porous polymer membrane used in EO pump is assumed to be a network of numerous pores with different radius and each pore is treated as a straight cylindrical microcapillary. It is considered that the pore radius in porous polymer membrane obeys Gaussian distribution which is consistent with the pore radius distribution in reality to a high degree [31,32,33].

The probability density function of Gaussian distribution is given as

(14) $$f\left(R\right)=\frac{1}{\sqrt{2\pi }\sigma }\exp\left[\frac{-{\left(R-v\right)}^{2}}{2\sigma^{2}}\right]$$

where *σ* is the variance and *ν* is the mean. It is a function of pore radius *R* of arbitrary single pore. Based on the probability density function *ϕ*(*R*), the mean value of single pore surface area is solved by

(15) $$A=P\int_{R_{\min}}^{R_{\max}}f\left(R\right)\cdot \pi R^{2}dR$$

where *R_min_* and *R_max_* are the minimum value and maximum value of pore radius, *P* is the modified coefficient [17]. As a result, the total number of pores for the given area *A_t_* is solved from the following expression

(16) $$N=\phi \frac{A_{t}}{A}$$

in which *ϕ* is the porosity of porous polymer membrane. Therefore, the total flow rate of power-law fluids through porous polymer membrane can be expressed as

(17) $$Q=\frac{NP}{\varepsilon }\int_{R_{\min}}^{R_{\max}}f\left(R\right)\cdot Q_{s}dR$$

in which *Q_s_* is the flow rate of arbitrary single pore which has been solved in Section 2.1 and *ε* is the tortuosity of porous polymer membrane.

## 3. Results and Discussion

The parametric values from practical applications are given as follows: the ion concentration of electrolyte solution is expressed as *n_∞_* = *N*_A_*c*, *N*_A_ = 6.02 × 10^23^/mol. The molar concentration *c* changes from 10^−2^ mol/m^3^ to 1 mol/m^3^ and is responsible for the change in electric charge density and then the Coulombic force. In addition, *e* = 1.6 × 10^−19^ C, *k_b_* = 1.38 × 10^−23^ J/(mol·K), *ρ_m_* = 1000 kg/m^3^, *m* = 9 × 10^−4^ Nm^−2^s*^n^*, *ε*_r_ = 80, *ε*_0_ = 8.85 × 10^−12^ C^2^/J∙m, *T* = 293 K. The dimensionless electrokinetic width, namely, the ratio of pore radius to the thickness of EDL *K = kR* changes from 10 to 50. The average value of single pore radius is *R* = 1.02 μm, the minimum value and maximum value of single pore radius are taken as 0.8 μm and 1.2 μm, respectively [17]. The applied voltage across porous polymer membrane varies from 10 V to 100 V, from which the electric field strength can be determined. The total area of porous polymer membrane *A_t_* = 2.5 mm^2^ and the thickness is given as *L* = 7–20 μm so that the electric field strength can be obtained from *E* = *V*/*L*. The membrane porosity is *ϕ* = 0.05–0.4 and the tortuosity is *ε* = 1.45 [17]. The value of zeta potential changes from 0.025 V to 0.2 V.

### 3.1. EOF of Power-Law Fluid in a Single Pore

At first to get a thorough understanding on EOF of power-law fluid in porous polymer membrane at high zeta potentials, the velocity profile, average velocity and flow rate in a single pore are presented for different fluid type, dimensionless electrokinetic width and zeta potential.

**Figure 2:**
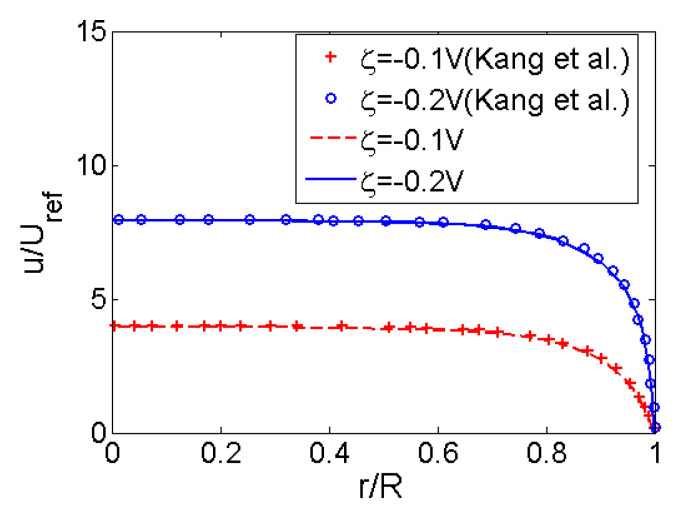
Comparison of the velocity profiles of EOF in the cross section of a single pore from the present model and the existing model of [30] at high zeta potentials where *n* = 1, *K* = 10 and the reference velocity *U*_ref_ = (*ε*_r_*ε*_0_/*m*)·*Ε*·(*k_b_T*/*e*).

In Figure 2, the numerical velocity profile for Newtonian fluid (*n* = 1) at high zeta potential solved from Equation (8) is compared to the existing result in [30]. The reference velocity is *U*_ref_ = (*ε*_r_*ε*_0_/*m*)·*Ε*·(*k_b_T*/*e*) which allows the effects of choosing different zeta potential stands out. It shows that the numerical result agrees well with the existing data even when the zeta potential is as high as |*ζ*| = 0.2 V, meaning that the numerical method adopted here is feasible.

**Figure 3:**
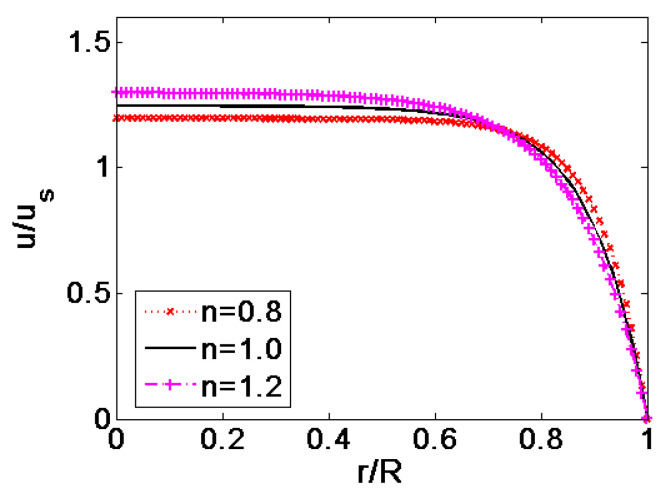
Comparison of the velocity profiles generalized by their respective average velocity at different flow behavior index *n* (*K* = 10, *ζ* = −0.1 V).

In Figure 3, the velocity profiles of shear thinning fluid, Newtonian fluid and shear thickening fluid are compared in the case of *K* = 10 and *ζ* = −0.1 V. To evidently exhibits the influence of flow behavior index on the velocity profile, the velocities are generalized its respective average velocity. As shown in Figure 3, the flow behavior index *n* plays major role on the patter of velocity profile. When the magnitude of flow behavior index increase from 0.8 to 1.2, namely when the fluid feature changes from shear thinning to Newtonian and shear thickening, the pattern of velocity profile exhibits plug-like shape at first, and then gradually changes to parabolic shape.

**Figure 4:**
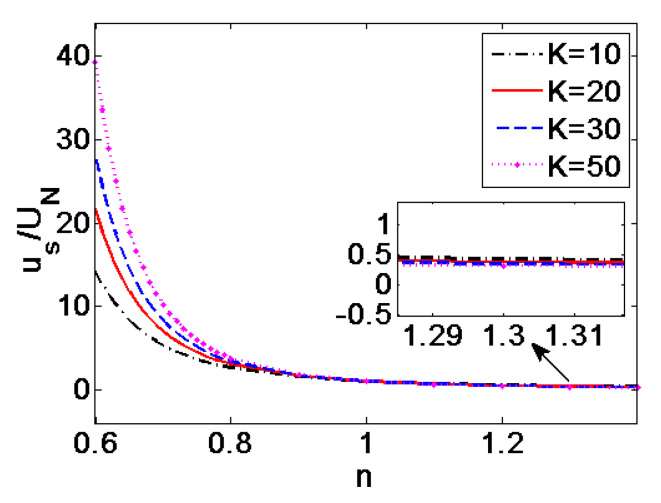
Variation of the average velocities generalized by the average velocity of Newtonian fluid (*n* = 1) with flow behavior index *n* at different dimensionless electrokinetic width *K* (*ζ* = −0.1 V).

In Figure 4, the average velocities of EOF in single pore generalized by the average velocity of Newtonian fluid (*n* = 1) are plotted at different dimensionless electrokinetic width *K* when *ζ* = −0.1 V. It can be seen from Figure 4 that when *n* < 1, i.e., in the case of shear thinning fluids, the average velocity shows more than ten times higher than the velocity of Newtonian fluid and shear thickening fluid. Moreover, the influence of dimensionless electrokinetic width *K* on velocity is evident. The velocity increases with *K*. As the flow behavior index changes to *n* > 1—namely, in the case of shear thickening fluid—the variation of velocity with *n* and *K* is slight, showing that the effects of *n* and *K* are weak. Thus, the velocity of EOF for shear thinning fluid is susceptible to the change in *n* and *K*, compared to that of shear thickening fluid.

**Figure 5:**
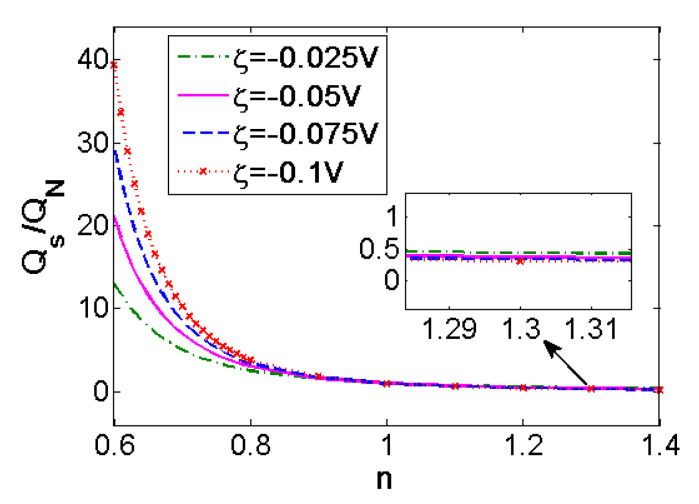
Variation of the flow rates generalized by that of Newtonian fluid (*n* = 1) with flow behavior index *n* at different zeta potential *ζ* (*K* = 50).

In Figure 5, the variation of the flow rate in single pore generalized by the flow rate of Newtonian fluid with the flow behavior index *n* is provided at different zeta potential when *K* = 50. The flow rate of shear thinning fluids is 10 times higher than that of Newtonian fluid and shear thickening fluids, and for greater value of |*ζ*|, the influence becomes more evident. The influence of zeta potential on the flow rate of shear thickening fluid is little. In practical applications, the EOF of shear thinning fluids can be adjusted by changing zeta potential.

### 3.2. EOF of Power-Law Fluid through Porous Polymer Membrane

**Figure 6:**
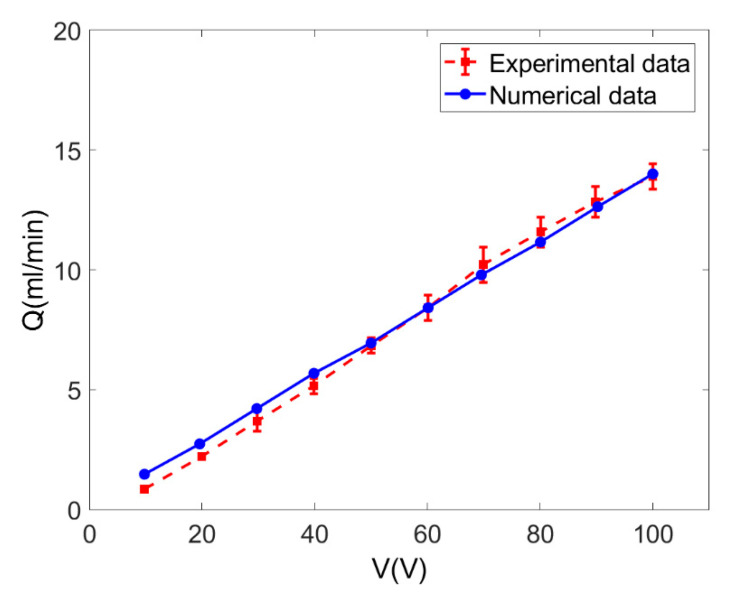
Comparison of the total flow rates obtained from the present model Equation (17) and the experimental data in [17] for Newtonian fluid (*c* = 0.01 mol/m^3^, *ζ* = −0.1 V).

Based on the discussion on EOF in a single pore, the performance of EO pumps operating power-law fluids is evaluated by computing the total flow rate of EOF in porous polymer membrane at different zeta potential, electrokinetic width and solution concentration. In Figure 6, the analytical flow rate for EOF of Newtonian fluid through porous polymer membrane obtained from Equation (17), is compared to the measured flow rate in [17] when *c* = 0.01 mol/m^3^, *ζ* = −0.1 V and more specific details can be found in Ref. [17]. The good agreement shown in Figure 6 validates the mathematical model presented for the EOF of power-law fluid through porous membrane. The mathematical model is able to fully describe the electroosmotic characteristics of power-law fluids through porous polymer membrane.

#### 3.2.1. Effect of the Applied Electric Voltage

**Figure 7:**
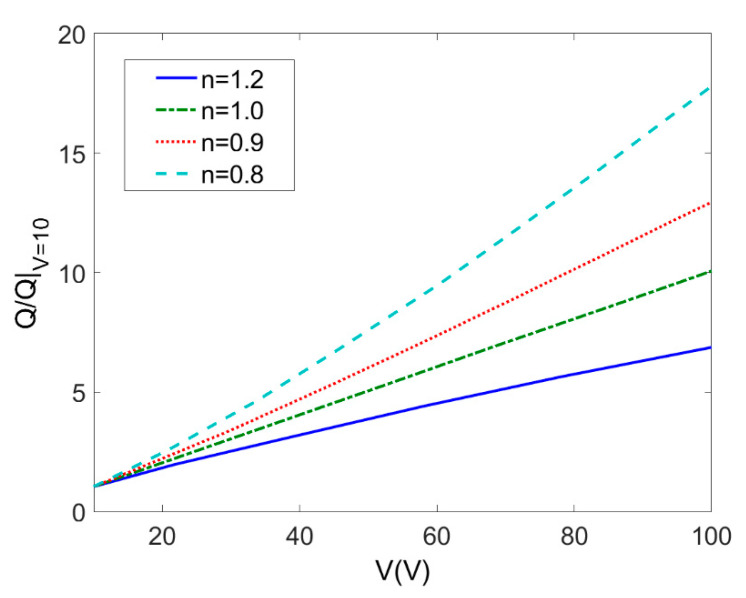
Variation of the total flow rates generalized by the total flow rate when *V* = 10 V with applied voltage at different flow behavior indices *n* (*c* = 0.01 mol/m^3^, *ζ* = −0.1 V, *L* = 20 μm, *ϕ* = 0.05).

The applied voltage plays a critical role on EOF of power-law fluids because the Coulombic force resulting from applying external electric voltage over porous polymer membrane is the driving force in EO pump applications. Figure 7 describes variation of the total flow rate with the applied voltage which ranges from 10 V to 100 V at different flow types (*c* = 0.01 mol/m^3^, *ζ* = −0.1 V, *L* = 20 μm, *ϕ* = 0.05). The total flow rate is generalized by the total flow rate when applied voltage is 10 V to show the influence of increase in applied voltage. The total flow rate of EOF through porous polymer membrane is increased for shear thinning fluids (*n* < 1) and it gets more evident as the flow behavior index *n* decreases. For shear thickening fluid (*n* > 1), the increasing rate of flow rate with the applied electric voltage is much less than that of shear thinning fluids.

#### 3.2.2. Effect of the Zeta Potential

**Figure 8:**
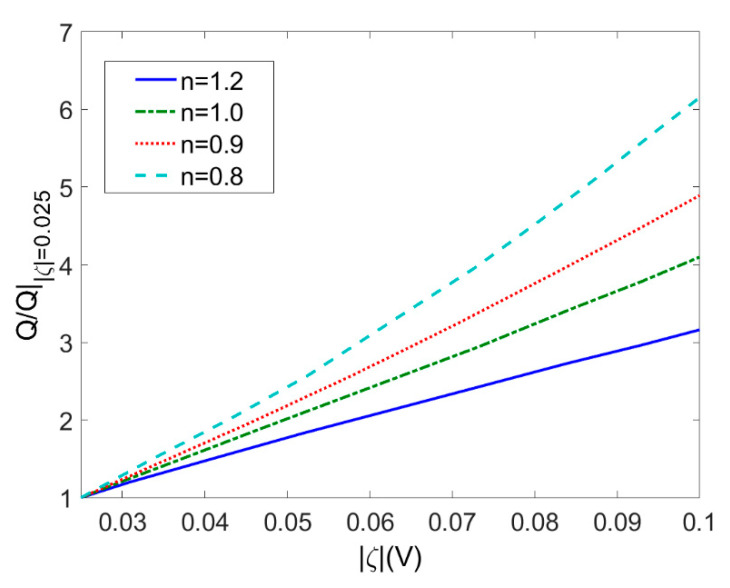
Variation of the total flow rates generalized by the total flow rate when *ζ* = −0.025 V with zeta potential *ζ* at different flow behavior indices *n* (*c* = 0.01 mol/m^3^, *V* = 10 V, *L* = 20 μm, *ϕ* = 0.05).

Figure 8 depicts variation of the total flow rate with zeta potential *ζ* for different flow behavior index *n* (*c* = 0.01 mol/m^3^, *V* = 10 V, *L* = 20 μm, *ϕ* = 0.05). The total flow rate is generalized by the total flow rate when |*ζ*| = 0.025 V. The increase in zeta potential exerts influence on the total flow rate which is especially significant for shear thinning fluids. It is notable that the total flow rate when |*ζ*| = 0.1 V is much more times higher than that when |*ζ*| = 0.025 V and thus one should have second thought of always assuming low zeta potential. It is reasonable to expect zeta potential has a remarkable influence on EO pumping performance.

#### 3.2.3. Effect of the Geometric Properties of the Membrane

**Figure 9:**
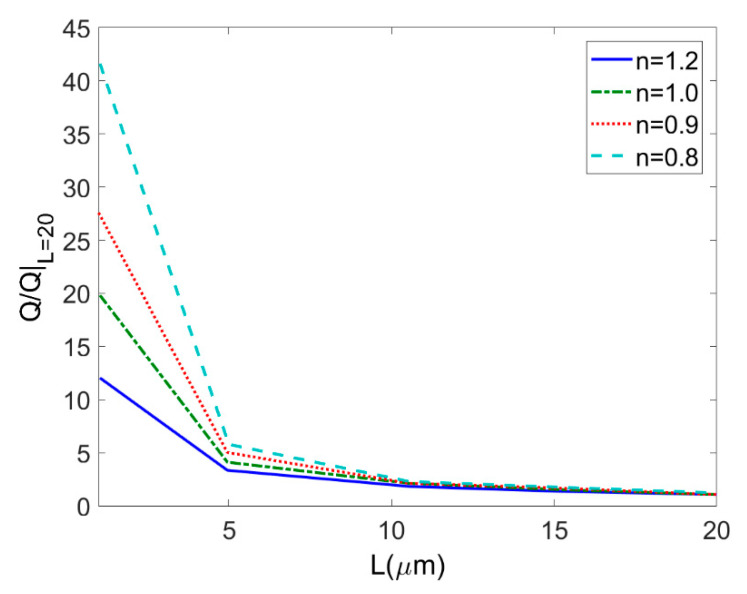
Variation of the total flow rates generalized by the total flow rate when *L* = 20 μm with membrane thickness at different flow behavior indices *n* (*c* = 0.01 mol/m^3^, *ζ* = −0.1 V, *V* = 10 V, *ϕ* = 0.05).

The effects of porous polymer membrane thickness *L* on the total flow rate at different types of fluids are shown in Figure 9 (*c* = 0.01 mol/m^3^, *ζ* = −0.1 V, *V* = 10 V, *ϕ* = 0.05). In Figure 9, the total flow rate for different types of power-law fluid is generalized by its respective total flow rate when the thickness *L* is 20 μm so that the influence of the increase in membrane thickness can be clearly shown. The total flow rates of power-law fluids through porous membrane decrease with the increment of membrane thickness. More specifically, the less the flow behavior index, the greater the decreasing rate of flow rate with the decrease of membrane thickness.

#### 3.2.4. Effect of the Electrolyte Solution Concentration

**Figure 10:**
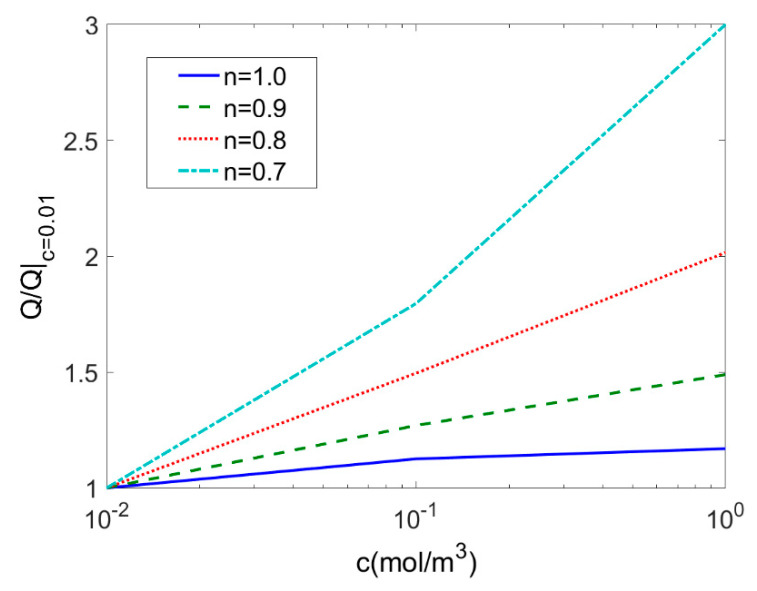
Variation of the total flow rates generalized by the total flow rate when *c* = 0.01 mol/m^3^ with electrolyte concentration at different flow behavior indices *n* (*ζ* = −0.1 V, *V* = 10 V, *L* = 20 μm, *ϕ* = 0.05).

The change in concentration of electrolyte solution leads to the change in ionic concentration and in further the dimensionless electrokinetic width *K*. Furthermore, the concentration of electrolyte solution can be readily controlled by adding or removing chemicals to solution. Correspondingly performance of EO pump can be modified. Therefore, Figure 10 shows variation of the total flow rate with electrolyte concentration ranging from 0.01 mol/m^3^ to 1 mol/m^3^ at different flow rate behavior *n* (*ζ* = −0.1 V, *V* = 10 V, *L* = 20 μm, *ϕ* = 0.05). For each type of fluid, the total flow rate is generalized by its respective total flow rate when the solution concentration *c* = 0.01 mol/m^3^. Since the variation of dimensionless electrokinetic width has insignificant influence on the average velocity for shear thickening fluids (*n* > 1) as shown in Figure 4 and hence in Figure 10 the variation of total flow rate with the electrolyte concentration is studied for Newtonian fluid and shear thinning fluids (*n* ≤ 1). It is found that the total flow rate increases with the electrolyte concentration and the increasing rate of flow rate gets higher as the flow behavior index decreases.

## 4. Conclusions

The study on EOF of power-law fluids through porous polymer membrane is summarized as follows:

- The characteristics of EOF for power-law fluids in a single pore is described in terms of velocity profile, average velocity and flow rate at first. The velocity profile shows more plug-like shape as the flow behavior index decreases (*n* < 1). A high zeta potential or a high dimensionless electrokinetic width can generate higher average velocity especially for shear thinning fluids in single pore.
- The mathematical model for EOF of power-law fluid through porous polymer membrane is presented by means of the assumption that porous polymer membrane has Gaussian distribution of pores. The performance of polymer porous membrane structure EO pump is evaluated from the perspective of the dependence of total flow rate on different parameters. The total flow rate of power-law fluid is increased with the increment of applied electric voltage, zeta potential, and concentration of solution. The influence of these parameters on total flow rate is much more evident for shear thinning fluids (*n* < 1) than that for shear thickening fluids (*n* > 1). Therefore, to attain high flow rate, high zeta potential, electrolyte solution with high ion density, thin membrane or shear thinning property of working liquid is desired in porous-structure EO pump applications.

The prediction for the electroosmotic flow of non-Newtonian fluid through porous polymer membrane may help enhance the performance of electroosmotic pump and other microdevices typically applied for the operation of bio-fluids, which are truly non-Newtonian fluid in nature.
